# How the COVID-19 pandemic impacts social scientific research on sustainability: questions of methodology, ethics and justice: comment on Santana et al. 2021

**DOI:** 10.1007/s11625-021-01066-y

**Published:** 2021-11-23

**Authors:** Danny Otto, Annegret Haase

**Affiliations:** Department of Urban and Environmental Sociology, Helmholtz-Centre for Environmental Research – UFZ, Permoserstraße 15, 04318 Leipzig, Germany

**Keywords:** Empirical research methods, COVID-19, Sustainability science, Justice

## Abstract

In a highly relevant contribution, Santana et al. (2021) outlined the challenges for qualitative enquiries during the pandemic. We agree that overcoming these challenges is very important since qualitative research is vital for understanding both the impacts of COVID-19 on human communities around the globe and its significance for sustainable futures. However, we argue that a more fundamental approach is needed to address problems within scientific organisations, thinking and practices that directly affect qualitative research capabilities. In this comment, we focus on justice, research organisation, the ways social scientists position themselves and changed understandings of social worlds.

## Introduction

The COVID-19 pandemic has deeply impacted scientific research in different ways. While it spurred pandemic-related research (e.g. Sachan 2020; Murray 2020), it slowed or stopped investigations in other fields. Regarding research practices, qualitative empirical research is certainly among those methodical approaches that were seriously hampered by regulations and restrictions during the pandemic. Santana et al. (2021) and others have addressed this issue and so far considered how research methods can be adapted to the restrictions and regulations related to COVID-19 (e.g. Vindrola-Padros et al. 2020). While we certainly agree that studying and overcoming these challenges is highly important, we aim to go beyond quick fixes for methods and raise issues that are more fundamental. We argue that changes in empirical research practices drastically effect the way social worlds are perceived through a methodological lens. Furthermore, this has crucial implications for the way research is organised, social justice and the (critical) self-positioning of social scientists. The aim of this comment is to add to the ongoing debates on the pandemic’s impacts on research by reflecting on these fundamental issues and their high relevance for sustainability research.

## Promising aspects and blind spots

The approach taken by Santana et al. (2021) shows several promising features. The authors are able to establish the importance of qualitative research for sustainability science and beyond. Since qualitative studies can provide “insights into the lived experiences of individuals and communities facing dramatic environmental changes”, they are apt to uncover unexpected themes and connections (Santana et al. 2021, p. 1062). These characteristics become particularly important during an unprecedented situation such as the COVID-19 pandemic that disrupts and questions the routines of entire societies and thereby seriously impacts sustainability efforts (Markard and Rosenbloom 2020; Kivimaa et al. 2021).

Based on this assumption Santana et al. (2021, p. 1063) convincingly outline physical, psychological and ethical challenges for qualitative research on sustainability caused by the pandemic and the associated restrictions (e.g. physical distancing, self-isolation). They furthermore list recommendations for overcoming these challenges, such as adapting qualitative research practices to digital environments or improving mental health measures for researchers and respondents. Several other articles have followed a similar pragmatic approach and adapted their qualitative research to the pandemic circumstances by collecting qualitative data in video conferences (e.g. Sah et al. 2020) or addressing vulnerable populations via online tools (Moyle et al. 2020).

This research orientation is highly useful and called for in the pandemic. It enables scientists to gather qualitative data and to gain much-needed insights into current societal dynamics. Articles address the feasibility of video conferencing for qualitative interviews (Moyle et al. 2020; e.g. Marhefka et al. 2020; Sah et al. 2020), online data collection for qualitative research (Deslandes and Coutinho 2020; Torrentira 2020) and how vulnerable people can be interviewed online (Dodds and Hess 2021). This research is crucial, but it fails to take advantage of the opportunity to reflect on the status quo and consider more sustainable research practices.

We argue that such quick fixes for empirical investigations will not suffice in the long run and take this as the entry point for our critical comment. Rather than offering an analysis of changes in underlying assumptions or transformations that are more fundamental to the research process, many studies have focused, much like Santana et al. (2021), on how social scientists can uphold empirical research activities during the pandemic. In the following, we want to outline some of the more profound effects of the pandemic on social scientific research. We think that it is vital to reflect on these issues to really understand how the pandemic is affecting our societies, lives, the ways we think, communicate and act—not to mention how we conduct social scientific research.

## Arguments on fundamental changes to social research

We found a small range of papers that critically debate the need for change in social science research and self-positioning due to COVID-19. Among other things, they argue that social science should understand the pandemic as a chance to critically assess and discuss research publication practices (“paperdemic”, “speed science”, see Dinis-Oliveira 2020; see also Sovacool et al. 2020). Yet too often reflections and conclusions about how to cope with the COVID-19 crisis as a researcher show that social science has so far responded to the crisis in a way that corresponds with the current paradigm: publish or perish.

One opportunity that the crisis offers social scientists, despite all odds and threats, is the chance to re-evaluate the relationship between research practices and inequalities. The pandemic has revealed and worsened inequalities, which prompted social scientists to acknowledge that they are not impartial observers and to reflect on their role in enacting, perpetuating and questioning inequalities (Boltanski 1987; Go 2013; Bourdieu 2013; Bhambra 2014). On the same note, Gilmore-Bykovskyj et al. (2021) remind us of the heightened risk of “under-includ[ing]” marginalised people due to “online research”, and Braun et al. (2020) stress the overall need for a responsible research and innovation approach to the “onlineification of research” that is inclusive, reflective and responsive to societal needs. This is especially relevant when addressing sustainability because the exclusion of underprivileged groups from debates and research on environmental issues has been repeatedly shown (e.g. Nagendra 2018; Sovacool 2014).

In addition to the problem of representation in research processes, social science needs to understand the pandemic as a collective crisis and consider how the scientists’ own involvement, affectedness and subjectivity impact the research process. In this situation characterised by rapid changes and existential threats, the validity of research is not only questioned because of the stark contrast between the social contexts before, during and after the pandemic, but also because of the instability and uncertainty that societies are faced with (Sovacool et al. 2020). Since social scientists are inevitably intertwined in this development, Stelson (2020) suggests reflecting on one’s own feelings and affectedness, the feelings of people who are part of the research field and on how personal situations may impact the analysis itself and the resulting conclusions.

Drawing on this literature and going beyond it, we want to stress three main arguments that we see as key challenges for a social scientific self-positioning debate that is needed in light of the pandemic:

## Social science has to rethink its approaches, empirical research foundations and methodologies

Even before the pandemic, the relationship between research methods and aspects of justice has been an ongoing topic of debate (Lyons et al. 2013; Koro et al. 2021). In light of the crisis, it becomes crucial to discuss how our methods can capture social worlds in a way that accounts for intersectional vulnerabilities and avoids the under-inclusion of marginalised groups (among others). How can we design our research so that it addresses sustainability in a clear and fundamental way and is, at the same time, socially responsible in the context of the pandemic? How can we address intersectional inequalities, particularly exposure to the virus and related restrictions, without stigmatising people? This is fundamentally important when we see that in some cities, for example, “poor” neighbourhoods or residential environments have been blamed for high infection rates or were even temporarily closed to protect others (e.g. Haase 2020).

We have to investigate how a shift in qualitative research towards digital tools for data gathering and to digitally available data overall (e.g. for content or discourse analysis) effects representational justice in research. Who is able to participate in this mode of empirical research? What happens if this trend persists beyond the pandemic, for instance because of the gains in time and cost efficiency experienced with online interviews compared to face-to-face interviews? Especially aspects such as the reduction of travel through online communication raises the question of how to strike a balance between sustainability and the requirements of research. Can we compare online interviews with those conducted face-to-face? How will this affect the development and application of methods that either cannot or can only barely be replaced by digital means (e.g. participatory observation, group discussions)? We also see a need for discussions on how to continue qualitative research if COVID-19 regulations are revoked (for instance because vaccinations have been rolled out or the development of medical treatment has progressed).

## Change in perception of social worlds

The crisis once more shows us that we are not doing research in a ‘neutral’ space. It stresses that we have not only to reflect upon how we are setting up, operationalising and carrying out our analyses. It is also necessary to think about the aspects of society we address with our research questions and perceive with our methods. Important societal issues might not only disappear from the social scientific view because of under-inclusion but also because other problems have taken centre stage during the pandemic. Therefore, it is necessary to monitor and discuss shifts in social science research topics. Furthermore, there is a dire need to ask how the pandemic-induced adaptations of (qualitative) research methods effect what we perceive of social realities. In particular, the lack of unmediated contact with respondents and their social and natural environment limits the ability of researchers to relate to their experiences (for instance, the risks and opportunities to adapt during the crisis or to sustainability efforts). Additionally, we have to consider how we handle the relationship between analytical and normative elements when dealing with societies in crisis. What is the role of social sciences during crises? This urges us to re-think how the expectation to provide clear assessments that serve as policy advice relates to the unavoidable normative and critical elements ingrained in these assessments.

## Research organisation and the position and role of social scientists

Lastly, we argue that the discussion of scientific research methods needs to go beyond the methods themselves and has to address the way research is organised. The inequalities within the field of (social) scientific research make it necessary to focus on the impact of the pandemic on research funding. It is unclear how the limitations to conduct some forms of empirical research will affect funding processes. Will scholars avoid submitting proposals involving qualitative research methods that rely on direct contact with respondents? Will funding agencies be reluctant to accept such proposals or introduce new requirements for planning such research projects (e.g. contingency planning, concepts for hygiene)? On a practical level, the successful conduct of international comparative qualitative (and quantitative) enquiries will be seriously affected, as possibilities to travel and meet in person will most likely continue to be limited. And, not least: How can we make sure that publication activities or the ‘presence’ generated through publications and scientific exchange consider the different pandemic restrictions and their impacts on the opportunities to do research and to participate in the discourse across the globe? If this is not considered appropriately, existing inequalities in the preconditions required to do research will be heightened, and social science also runs the risk of losing track of many facets and realties of social worlds during the pandemic through the limited presence or absence of a part of its community.

Having outlined these challenges, we would like to emphasise that it is our deep belief that social scientists will be hard pushed to deal with these questions (and more) amidst a global crisis while struggling to keep up their publication activities, adhere to deadlines and secure funding for upcoming projects. Yet we nevertheless see the dire need for global discussions among social scientists as the pandemic fundamentally affects research perspectives, the quality of results and the way we are able to perceive social worlds through our methods. We should not just learn how to best adapt to the conditions of crisis, but also try to better understand that the crisis requires us to reposition ourselves and the way in which we organise, operationalise and carry out our research.
